# A Unique Presentation of Cutaneous Diffuse Large B-Cell Lymphoma

**DOI:** 10.1155/2020/8310602

**Published:** 2020-04-07

**Authors:** Mary Awad, Erik Holzwanger, Sandeep Jubbal

**Affiliations:** University of Massachusetts Medical School, Worcester, MA, USA

## Abstract

Cutaneous B-cell lymphomas (CBCL) are rare heterogeneous neoplastic diseases composing about 22.5% of all cutaneous lymphomas. These diseases can be divided into primary and secondary cutaneous variants with primary cutaneous B-cell lymphoma (PCBCL) divided into three distinct entities including primary cutaneous marginal zone lymphoma, primary cutaneous follicle center lymphoma, and primary cutaneous diffuse large B-cell lymphoma, leg type (PCDLBCL, LT). Secondary cutaneous diffuse large B-cell lymphoma (CDLBCL) and PCDLBCL, LT are more aggressive neoplasms compared to the aforementioned CBCL with survival rates of 37% and 50% after 5 years, respectively. CDLBCL can present as cutaneous or subcutaneous nodules, papular lesions, or indurated plaques. Here, we present a case of CDLBCL of an 88-year-old female that was mistaken for lower extremity cellulitis with phlegmon. Our patient failed two courses of antibiotic therapy as an outpatient and received a third as an inpatient before a cutaneous biopsy clinched the diagnosis.

## 1. Introduction

Diffuse large B-cell lymphoma (DLBCL) comprises one-third of all non-Hodgkin's lymphomas (NHL). DLBCL is composed of multiple diverse neoplasms involving both primary extranodal and nodal types. Epidemiologically, DLBCL is most common in Caucasians and men and the incidence increasing with older age. Of the extranodal subtypes, gastrointestinal, head and neck, and skin and soft tissue comprise the majority of primary sites. The distinction between primary cutaneous diffuse large B-cell lymphoma (PCDLBCL) and DLBCL with secondary cutaneous involvement is based on extracutaneous involvement at the time of diagnosis. PCDLBCL can metastasize to extracutaneous sites, as is seen in 40% of patients with PCDLBCL, leg type (PCDLBCL, LT), often making it difficult to establish a primary cutaneous diagnosis [1]. Prognosis of DLBCL is variable based on subtype with 5-year survival rates ranging from 30–80%; however, prognosis of secondary cutaneous DLBCL and PCDLBCL, LT are both poor with 5-year disease specific survival rates at 37% and 50%, respectively [2]. It is therefore critical to diagnose cutaneous diffuse large B-cell lymphoma (CDLBCL) early on in its pathogenesis [3].

## 2. Case

**Figure 1:**
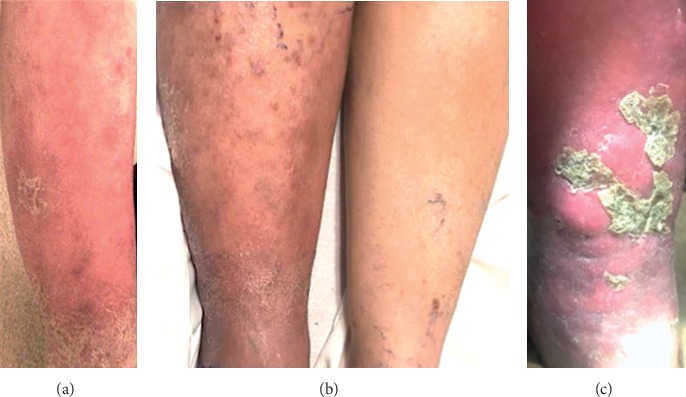
(a) Photograph of right lower extremity anterior view taken 4 days prior to admission following 10 days of cephalexin therapy. (b) Photograph of bilateral lower extremities anterior view taken on hospital day 4; on day 4 of cefazolin therapy prior to biopsy and diagnosis. (c) Photograph taken on hospital day 10 after 3 failed treatments of antibiotic therapy and after diagnosis of DLBCL.

An 88-year-old woman with a history of type II diabetes mellitus, chronic deep venous thrombosis (DVT) of right femoral vein, and atrial fibrillation on coumadin presented to her primary care physician (PCP) with right lower leg extremity swelling, erythema, and pain. A week prior to presentation, there was development of a sore on her right posterior ankle that began to crust over. The patient's leg became increasingly edematous, painful, warm, and erythematous throughout the week. Her primary care physician prescribed cephalexin for 10 days followed by doxycycline for four days. She continued to have progression of her symptoms while on these antibiotics (Figure 1(a)). She then developed intermittent purulent drainage from the sore behind the ankle concurrently with severe pain radiating down the leg (Figure 1(c)). She was then hospitalized and assessed for a deep soft tissue infection, abscess, and deep vein thrombosis. Cefazolin was given with subsequent improvement in the degree of erythema, though she continued to endorse significant pain. As the erythema receded to the mid-shin, the patient had persistent scattered papules and macules along the upper calf (Figure 1(b)). Blood cultures were negative and over the course of her illness, she remained afebrile without night sweats or chills. Laboratory workup revealed an elevated CRP (11.4 mg/L), which was felt to be secondary to an active infection. At presentation, a complete blood count with differential was within normal limits (white blood cell count 6.0 thousand cells/*μ*L). On hospital day 3, she underwent a CT lower extremity with contrast due to continued pain, which was significant for diffuse heterogeneity in the calf muscle, notably a 1.8 × 1.3 × 3.8 cm lesion in the gastrocnemius without evidence of a drainable fluid collection or air within the soft tissue. The leading differential at this time was myositis with phlegmon leading to persistent pain and continued erythema and edema. She proceeded to have a skin biopsy for culture and histochemistry, which displayed an atypical mononuclear infiltrate, and previous imaging was reinterpreted as possible malignancy. Immunohistochemical staining done was negative for CD10 and positive for CD20, MUM1, BCL-2, and BCL-6 consistent with primary cutaneous diffuse large B-cell lymphoma, leg type; however, secondary cutaneous nongerminal DLBCL was not ruled out.

Of note, during hospitalization LDH was found to be elevated to 506 U/L. Following discharge, there was an overall increase in LDH value with slight fluctuations from 694 U/L to 616 U/L and ultimately reaching a maximum value of 737 U/L when last assessed. Patient's complete blood count remained unremarkable throughout hospitalization besides for mild normocytic anemia (hemoglobin approximately 11.5 g/dL). CT abdomen and pelvis with contrast revealed an enlarged right inguinal lymph node 1.6 × 1.8 cm. There was no intra-abdominal or retroperitoneal lymphadenopathy. CT chest with contrast did not show new nodules nor did it show mediastinal or hilar adenopathy. PET/CT scan performed 2 weeks after discharge and 1 month following presentation showed extensive intensely FDG-avid muscular, soft tissue, and dermal lymphomatous involvement in lower extremities, right greater than left. There were additionally discrete soft tissue nodules and lymph nodes in the lower extremities, left buttock, right inguinal region, and right external iliac. The patient was diagnosed with advanced stage IV nongerminal DLBCL. The patient was later started on rituximab to polychemotherapy (R-CHOP) but was transitioned to hospice care after developing sepsis, DIC, and a new DVT.

## 3. Discussion

DLBCL typically presents in males in the 7^th^ decade of life with a median age of 64 years old, though DLBCL can present at any age, accounting for 20% of NHL in children under 14 years old [4]. In a study comparing cutaneous lymphomas, it was found that 22.5% of cases of cutaneous lymphoma were of B-cell lymphomas with 44.4% constituting primary cutaneous disease [5]. Primary cutaneous lymphomas are divided into 3 subtypes according to the 2008 World Health Organization-European Organization for Research and Treatment of Cancer (EORTC) including primary cutaneous marginal zone lymphoma, primary cutaneous follicle center lymphoma, and primary cutaneous diffuse large B-cell lymphoma, leg type [6].

PCDLBCL, LT in contrast to the aforementioned CBCL and other DLBCL commonly affects females and has a poor prognosis. The median age for PCDLBCL, LT at presentation is 70–82 years old [1, 6]. Most cases involve the lower legs; however, around 10% of cases can involve other cutaneous sites and extracutaneous dissemination is common. Most cases are MUM-1/IRF-4, BCL-2, and BCL-6 positive as was seen with our patient [6].

A study assessing presentations of DLBCL secondary cutaneous disease found that secondary cutaneous disease most commonly involves the legs and most frequently presents as cutaneous nodules, but in 16% of cases, it presents with papular lesions, as was seen with our patient [3]. There is scarce literature detailing the course of secondary cutaneous DLBCL. The prognosis and clinical course is much more aggressive in secondary cutaneous DLBCL compared to the indolent PCBCL in what has been reported. A patient diagnosed with secondary cutaneous DLBCL qualifies for stage IV disease per Ann Arbour staging giving an unfavorable diagnosis to those with the disease. Other unfavorable factors include age > 60, ECOG > 2, involvement of more than 2 extranodal sites, and elevated LDH [4].

Our patient's case was complex due to her comorbidities, partial response to antibiotic therapy, and lack of constitutional symptoms which led to a difficult diagnosis. Initially, we believed her underlying type II diabetes mellitus, peripheral vascular diseases, and chronic lower extremity edema predisposed her to an infectious etiology. Therefore, we continued to treat as if this patient had a cellulitis with phlegmon in the setting of supportive radiographic evidence. Ultimately, minimal improvement in symptoms led to a biopsy leading to a diagnosis of CDLBCL. This case highlights the difficulty in diagnosing patients with CDLBCL given the rarity and unusual presentation of this illness. At the time of diagnosis, our patient had extracutaneous involvement including the gastrocnemius and, on PET-scan, surrounding lymph nodes therefore limiting our confidence in diagnosing primary cutaneous disease.

Survival rates for CDLBCL have improved with the addition of rituximab to polychemotherapy (R-CHOP); however, up to 40% of disease becomes refractory to this treatment or relapses [4]. Unfortunately, our patient was unable to complete R-CHOP therapy after developing DIC, sepsis, and a new DVT. Given the late age of onset of this disease, aggressive polychemotherapy may not be feasible, and therefore more targeted or localized therapies are required. Exciting developments in CD19 targeted chimeric antigen receptor T-cell (CAR-T) therapy show promise in treating B-cell malignancies expressing the CD19 transmembrane glycoprotein, as is seen in many DLBCL. However, these treatments are costly and can have consequential side effects such as cytokine release syndrome and neurotoxicity [7]. Gaps do remain in our understanding of this disease process, specifically in secondary cutaneous disease. Research must focus on understanding the microenvironment of this neoplasm as a means of better targeting the cells driving this process.
